# P-955. Single vs. Multiple-Fellow Triaging Systems in a Large Academic Infectious Disease Service – A Quality Improvement Project

**DOI:** 10.1093/ofid/ofae631.1145

**Published:** 2025-01-29

**Authors:** Francisco J Machiavello Roman, Marwan M Azar

**Affiliations:** Yale School of Medicine, New Haven, Connecticut; Yale University, New Haven, Connecticut

## Abstract

**Background:**

The Yale New Haven Hospital (YNHH) is a large academic center with 3 Infectious Diseases (ID) consult services (General, Heme-Onc, and Transplant ID), each staffed by 1 fellow and attending physician. In the traditional consult triaging system, one fellow receives all the consults, often ≥15/day, and assigns them to each ID service. Fellows alternate the triaging duty during the week, resulting in 2-3 triaging days/week/fellow. Our program review identified that this system was a significant source of burnout and contributed to prolonged work hours, which prompted a QI project to address these concerns.

**Figure:**
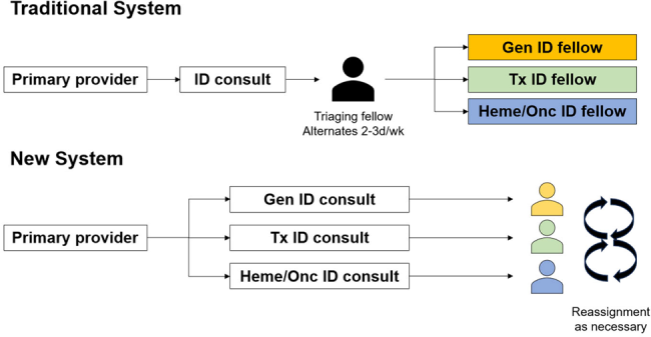

Traditional and New Proposed Triaging Systems at YNHH

**Methods:**

We designed an 8-question survey to assess the effect of triaging on the fellows’ workload, performance and mental health (anxiety and burnout). 2 interventions changed the consult triage workflow: i) The ID consult order on the electronic medical record was reformatted to require that primary providers select an ID subspecialty service, and ii) We eliminated the traditional triage role by asking the YNHH call center to route service-specific consults to the appropriate ID fellow (Figure 1). We conducted the pre-intervention survey in November 2023. The interventions took place in December 2023. The survey was repeated in February 2024.

**Figure:**
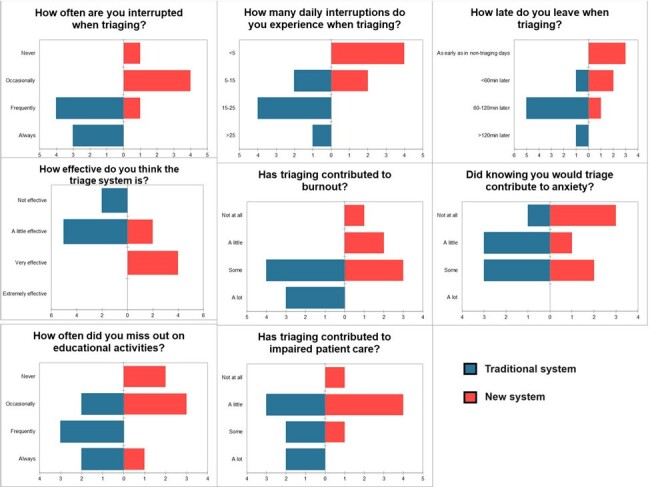

Pre and post-intervention surveys

**Results:**

7 (100%) and 6 fellows (86%) completed the pre and post-intervention surveys (Figure 2). In the pre-intervention survey, 57% reported frequently experiencing interruptions, and 57% reported an average of 15-25 interruptions/day. 86% stated that the traditional system contributed to a little or some anxiety, and 100% that it caused some or a lot of burnout. 57% replied that it contributed to some or a lot of impaired patient care.

In the post-intervention survey, 17% reported frequently experiencing interruptions, and 67% reported an average of < 5 interruptions/day. 50% stated that the new system contributed to a little or some anxiety, and 50% said that it caused some or a lot of burnout. 17% replied that it contributed to some or a lot of impaired patient care.

**Conclusion:**

A switch from a single-fellow to a multiple-fellow triaging system in our ID division led to decreased interruptions, decreased feelings of anxiety and burnout, and reduced perceptions of impaired patient care.

**Disclosures:**

**All Authors**: No reported disclosures

